# Observing the behavioural effects of methylphenidate in children and adolescents with ASD-ADHD dual diagnosis: A mini review

**DOI:** 10.3389/frcha.2023.1052115

**Published:** 2023-03-17

**Authors:** Danilo Dimitri, Giuliana Delia, Maurizio Arduino, Nazarena Turco, Franco Fioretto

**Affiliations:** ^1^ Department of Psychology, University of Turin, Turin, Italy; ^2^ Department of Child Neuropsychiatry, A.S.L. CN1- Mondovì, Neuropsichiatria Infantile, Mondovì, Italy; ^3^ Center for Autism and Asperger’s Syndrome, A.S.L. CN1, Mondovì, Italy

**Keywords:** attention deficit hyperactivity disorder, ADHD, autism spectrum disorder, ASD, ASD-ADHD, comorbidity, methylphenidate, children and adolescents

## Abstract

**Research aim:**

The aim of this study is to focus on the main neurophysiological aspects of attention-deficit/hyperactivity disorder (ADHD) and autism spectrum disorder (ASD) and the current pharmacological treatment used for the management of hyperactivity and attention deficits in children aged 6-20 years with a diagnosis of ASD, not associated with other genetic or epileptic disorders, such as Fragile X Syndrome (FXS), Tuberous Sclerosis, Kleefstra Syndrome or Angelman Syndrome.

**Methods:**

This mini review was conducted according to the *P*.I.C.O. model and according to the PRISMA guidelines. The keywords used were: *autism spectrum disorder*; *attention deficit hyperactivity disorder*; *attention deficit disorder*; *methylphenidate*; *ritalin*; *ADHD*; *youth autism*; *childhood autism*; *childhood autism spectrum disorder*; *adolescent autism*. The strings produced were compared and selected by a third independent clinician. The PubMed and PsycArticles search yielded a total of 3,200 articles. For their inclusion, the 3,200 articles were examined by two clinicians who ultimately selected 28 (15 clinical trials and 13 reviews/meta-analyses) articles analysed according to their consistency with the inclusion and exclusion criteria.

**Conclusions:**

Three main aspects emerged from the review: (1) According to the existing literature, new randomized controlled trials are needed to ensure a better understanding of the most effective drug treatments for dual-diagnosed ASD-ADHD patients and of the related behavioural effects. Currently, the use of drugs varies depending on psychiatric comorbidity, symptoms, age and gender and there is no univocal reference therapy; (2) Methylphenidate (MPH) has currently been shown to be the most suitable drug for the treatment of hyperactivity and inattention in individuals diagnosed with ASD and ADHD; (3) There is a need to create and evaluate appropriate tests to analyse more specific patterns of behaviour presented in the two conditions.

## Introduction

Attention-deficit/hyperactivity disorder (ADHD) and autism spectrum disorder (ASD) are two of the most common neurodevelopmental disorders (1). ASD is defined by persistent social and communication deficits, as well as restricted, repetitive, and stereotyped behaviours. This condition has an overall prevalence of between 1% and 3% in the paediatric population. ADHD is characterised by an attention deficit paired with hyperactivity/impulsivity, and it has an estimated prevalence ranging from 2% to 7% (2, 3).

An increase in both ASD and ADHD diagnoses has been registered in the last decade, owing to a change in the clustering method used. Before 2013, the Diagnostic and Statistical Manual of Mental Disorders 4 (DSM-IV) defined ADHD and ASD as two separate disorders and did not consider their possible co-occurrence (4). With the introduction of the DSM-5, it has become possible to pay more attention to the potential comorbidity between the two conditions (5). Moreover, according to the new guidelines for the diagnosis of ADHD and ASD, Asperger's syndrome, and pervasive developmental disorder-not otherwise specified (PDD-NOS), although labelled and diagnosed as separate diseases until 2013, they have now been incorporated into the macro-category of ASD.

The connection between ADHD and ASD has been widely analysed, leading to the proposal of three different comorbidity scenarios: (1) impulsivity that leads to difficulty in the comprehension of social cues; (2) hyperactivity in connection to stereotyped and repetitive behaviours; and (3) co-presence of attention deficits, difficulty in understanding social cues, and low verbal intelligence quotient (IQ) (6). This indicates an increasing interest in the underlying behavioural mechanisms of the two pathologies, which would allow better treatment of ADHD and ASD as coexisting conditions.

The reliability of Methylphenidate (MPH) for the treatment of children with ASD and comorbid ADHD has been discussed over the past decades, and its employment has been hampered by conflicting data. Early studies, dating back to the 1970s, indicated limited efficacy of MPH and significant side effects, together with poor tolerability and frequent aggravation of behavioural and social issues (7). They also underlined the possible iatrogenic effects of MPH, varying from irritability and aggressiveness to increased stereotypies. Additional studies from the 1980s confirmed the side effects linked to MPH use and described a frequent manifestation of motor restlessness, increased stereotyped movements, and psychotic symptoms (8). However, in contrast to what was claimed, more recent investigations suggest that the use of stimulant medications might be beneficial in reducing the symptomatology in children with an ASD-ADHD diagnosis who show typical ADHD symptoms. Stimulant treatment has been associated with an increased attention span and reduction in hyperactivity and impulsiveness (7).

The main aim of this qualitative review was to provide an overview of the most relevant randomised controlled trials, systematic reviews, and meta-analyses on this topic, as well as to describe the effects of MPH in children with an ASD-ADHD dual diagnosis.

## Methods and search strategy

The use of MPH in the clinical practice for the treatment of ASD patients presenting with comorbid ADHD was analysed to its efficacy in treating hyperactivity and attention deficits. As this was not a meta-analysis, the transcribed data were purely descriptive. This systematic review was conducted according to the P.I.C.O. model (9):

P (Patient): Children aged 6–20 years with a diagnosis of ASD in comorbidity with ADHD.
I (Intervention): An analysis of the available literature on the use of MPH paired with a traditional clinical rehabilitative treatment of the patient(s).
C (Comparison): Traditional treatment of the patient(s) without additional use of MPH.
O (Outcome): Evaluation and analysis of the efficacy of MPH in the reduction of ADHD symptoms, specifically inattention-impulsivity and motor restlessness.

The review of the available literature considered all articles and reviews which included a sample of children and adolescents aged 6–20 years published between 2010 and 2021 in English. The exclusion criteria were applied to single-case studies, studies that examined ASD patients with an IQ < 70, patients aged <6 or <20 years, and studies that mentioned a comorbidity with epilepsy, Tourette syndrome, Rett syndrome, Fragile X syndrome, and/or Trisomy 21.

This systematic mini review was conducted according to the PRISMA guidelines (9). The keywords and query strings were identified by two independent clinicians. The strings produced were compared and selected by a third clinician. PubMed and PsycArticle were used as search engines by applying the following query strings.

[Autism spectrum disorder(tiab)] **AND** (attention deficit hyperactivity disorder) **AND** (methylphenidate) **AND** (“Meta-Analysis"[ptyp] **OR** systematic[sb]**OR** systematic*[title] **OR** meta-anal*[title])
*[Autism spectrum disorder(tiab)] **AND** (attention deficit hyperactivity disorder) **AND** (methylphenidate)*
*((childhood autism) **OR** (autism spectrum disorder childhood) **OR** (adolescent autism) **OR** (adolescent asd)) **AND** (adhd) **AND** ((methylphenidate) **OR** (ritalin))*
*((childhood autism) **OR** (ASD) **OR** (childhood autism spectrum disorder) **OR** (adolescent autism) **OR** (youth autism)) **AND** (ADHD) **OR** (attention deficit disorder)) **AND** ((methylphenidate) **OR** (ritalin)).*

Finally, all references mentioned in the articles not directly detected in the database search were analysed and incorporated in the final article count, if consistent with the inclusion and exclusion criteria.

**Figure 1:**
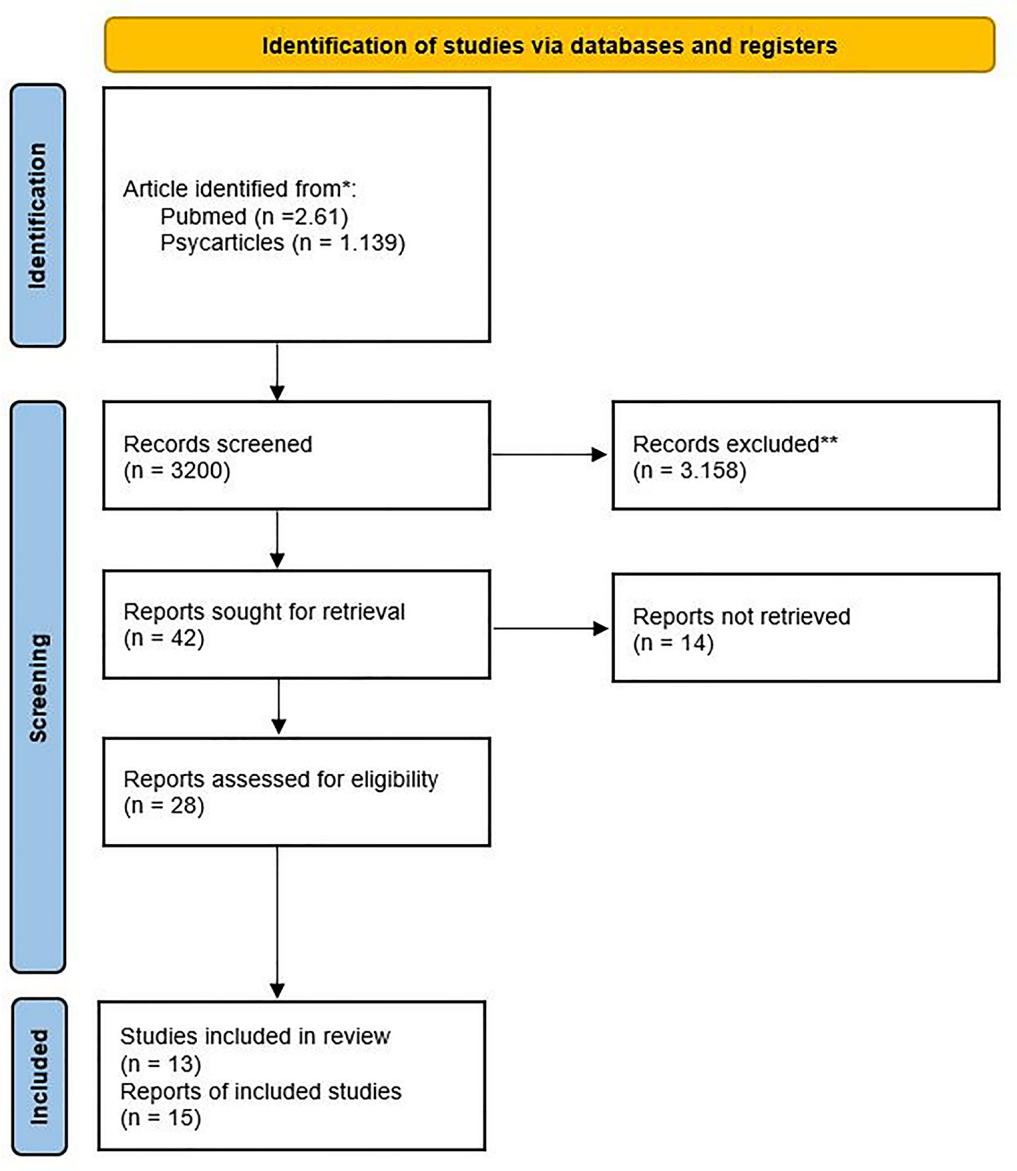
PRISMA 2020 flow diagrams for reviews which included searches of databases. * Consider, if feasible to do so, reporting the number of records identified from each database searched (rather than the total number across all databases/registers). ** If automation tools were used, indicate how many records were excluded by a human and how many were excluded by automation tools.

A total of 3.200 articles were retrieved from the database search. For the inclusion of the suitable articles, their titles were examined by two clinicians who ultimately selected 42 articles, which were further analysed according to their consistency with the inclusion and exclusion criteria. 28 articles were included in the final paper count and divided into two categories: clinical trials (15 articles) and reviews/meta-analyses (13 articles). The research process is presented in a summary PRISMA 2020 flow diagram (Figure 1) (10).

## Results

Three main elements were found: (1) the efficacy of MPH in reducing the typical symptoms of ADHD in children diagnosed with ASD-ADHD, (2) the tolerability of the drug and the reduced side effects, and (3) the characteristic pathophysiology of subjects diagnosed with ASD-ADHD, which allowed us to understand the action of MPH on the networks involved in the disease.

### Efficacy of methylphenidate

From the literature review it emerges that while patients with ASD appear to be prescribed different medications based on their characteristics, ranging from antipsychotics to stimulants, antidepressants or anxiolytics, patients diagnosed with ADHD generally receive a more standard prescription of psychostimulants (MPH in 49% of cases), followed less frequently by melatonin and anxiolytics (11). These data underline the efficacy of stimulant therapy for the treatment of ADHD, but also highlight a greater heterogeneity in drug prescribing and the lack of a specific treatment protocol, which currently differs from country to country (11, 12). This hinders the formulation of unique protocols. To date, several experts agree on the importance of performing a blood screening prior to drug treatment, which should include a complete blood cell count (CBC), basic metabolic panel (BMP), kidney function tests, and electrocardiogram (ECG) (13).

As for the efficacy of MPH, results from seven clinical trials examined are shown in Table 1. Several studies have demonstrated significant efficacy of MPH on ADHD symptoms in children diagnosed with pervasive developmental disorders (PDD) or ASD, particularly with regards to hyperactivity and inattention (14).

**Table 1:** Studies focused on MPH efficacy: This table shows the significant clinical studies focused on MPH use for the treatment of ADHD symptoms in subjects aged between 5 and 21 years old.

N.	Author(s)	Years	Sample (N)	Age	Assessment tools ASD-ADHD	Test Evaluation of effectiveness	Level of the disorder	MPH dosage	Drug type	Behavioral effects
1	Pearson et al.	2013	24	7-12	- ADI-R - DSM-IV-TR (APA 2000) - ADOS - DICA-IV - CPRS-R - CTRS-R	- CPRS-R - CTRS-R - CGI - SNAP-IV - ACTeRS - ABC - VAS - PDR	HFA - Autistic disorder (n=19)	- Low dose 0.21 mg/kg 0.14 mg/kg - Medium dose 0.35 mg/kg 0.24 mg/kg - High dose 0.48 mg/kg 0.27 mg/kg	Extended release	Significant improvements in cognitive skills: sustained and selective attention; impulsivity; inhibition
2	McCracken et al.	2014	64	5-14	- DSM-IV-TR (APA 2000) - ADI-R - CGI-S-ADHD - SNAP-IV	- CGI - ABC - SNAP-IV	HFA - Asperger's disorder (n=5) - PDD-NOS (n=14) - Autistic disorder (n=47)	- Low dose 0.125 mg/kg - Medium dose 0.25 mg/kg - High dose 0.5 mg/ kg	Extended release	Improvements in hyperactivity and impulsivity for all genotypes
3	Kim et al.	2017	69	5-17	- DSM-IV-TR (APA 2000)/DSM-5 (APA 2013) - K-SADS-PL - CGI-S-ADHD	- ADHD RS-INV - CGI-I - ABC - RISC-K	HFA	- Low dose 5-20 mg/ kg/die - Medium dose 5-40 mg/ kg/die - High low dose 5-10 mg/ kg / die	Extended release	Clinically significant improvements in ADHD symptoms in the medium-dose group
4	Scahill et al.	2017	60	5-14	- ADI-R - PTP - ABC Hyperactivity subscale - SNAP-IV - SLOSSON IQ - VINELAND	- CGI - ABC - Parent target problem - Parent target problem rating	HFA	- Low dose 0.125 mg/kg - Medium dose 0.25 mg/kg - High dose 0.5 mg/kg	Instant release	Generalized improvements in executive functions, depending on dosage
5	Peled et. al.	2019	40	6-18	- DSM-5 - ADOS - CARS	- MAXO CPT - Attention -Timing Impulsivity - Hyperactivity	HFA	Dose 10 mg/kg	Instant release	Significant improvements in processing speed (PS)
6	Pearson et al.	2020	24	7-12	- ADI-R - ADOS - DSM-IV-TR - DICA-IV (ADHD) - CPRS-R E CTRS-R	- MFT - CPT - SCT - PSI - DGT - MFFT - SST	HFA - Autistic disorder (n = 19) - Asperger's disorder (n = 3) - PDD-NOS (n = 2)	- Low dose 0.21 mg/kg - Medium dose 0.35 mg/kg - High dose 0.48 mg/kg	Instant release	Improvement of activities that exploit the cognitive components of sustained and selective attention.
7	Ventura et al.	2020	80	6-21	- DSM 5 (APA 2013) - CPRS-R - ADOS-2 - ADI-R - WPPSI-III - WISC-IV - ASDI - LEITER-R - CBCL	- C-GAS - CGI	HFA	Dose 0.3-0.5 up to a maximum of 1 mg/kg	Instant or extended release	Significant reduction in the severity of symptoms, improvement in overall functioning

ABC, Aberrant Behavior Checklist; ACTeRS, ADD-H Comprehensive teacher's Rating Scale, Second Edition; ADHD RS-INV, ADHD Rating Scale, Investigator Version; ADI-R, Autism Diagnostic Interview-R; ASDI, Autism spectrum Diagnostic Interview; CGI-S-ADHD, Clinical Global Impressions—Severity for ADHD; CARS, Childhood Autism Rating Scale; CBCL, Clinical observation and children behavioral checklist; Clinical Global Impression (CGI)-Severity; (CGI-P) C-GAS, Clinical global assessment scale; CPRS-R, Conners Parent Rating Scale; CPT, Continuous Performance Test; K-SADS-PL, Diagnostic interview for the evaluation of psychopathological disorders in children and adolescents; ADOS/ADOS-2, Diagnostic Observation Schedule; DGT, Delay of Gratification Task; DICA-IV, Interview for Children and Adolescents-IV; Leiter-R, Leiter International Performance Scale-Revised; DSM 5, Mental Health Diagnostic Manual 5; MFFT, Matching Familiar Figures Test; CPT, MAXO Continuous Performance Test; MFT, National Marital and Family Therapy Examination; PTP, Parent target problem; ABC, Parent/ Teacher Aberrant Behavior Checklist; Pervasive developmental disorder PDD-NOS; PDR, Physician's Desk Reference; RISC-K, Response Impressions and Side Effects Checklist-Kids; SLT, Selective listening task; SCT, Speeded Classification Task; Slosson Intelligence Test-4th Edition (Slosson-QI); SST, Stop Signal Task; SNAP-IV, Teacher and Parent Rating Scale; Vineland Adaptive Behavior Scales (Vineland); WPPSI-III, Wechsler Preschool and PrimaryScale of Intelligence-Third Edition; VAS, Visual Analog Scale; WISC-IV, Wechsler Intelligence Scales for Children-Fourth Edition.

The results of the meta-analysis of Reichow et al. and Sturman et al. suggest that MPH is effective in treating ADHD in children with PDD/ASD (14, 15).

The articles showed a general homogeneity in the diagnostic tools implemented for the assessment of both ASD and ADHD. All studies reported little or no influence of MPH on motor stereotypes and social behaviour. However, a meta-analysis by Sturman et al. demonstrated that a treatment with MPH could reduce hyperactivity in children with ASD and ADHD for a short period of time (14). This event was further confirmed by the results obtained from several behavioural rating scales compiled by teachers and caregivers. Specifically, 4 studies used the Conners Global Impression (CGI) index to assess patient improvements, which measures hyperactivity using the Conners Scale (CSR-S) (3, 13, 16, 17). Overall, all 6 clinical trials showed improvement in inattention, repetitive behaviours, and hyperactivity in children treated with MPH. However, this improvement appears to be directly related to dosage. Data from the meta-analysis of Sturman et al. documented that with methylphenidate doses of 0.43 mg/kg/dose to 0.60 mg/kg/dose, a significant and clinically relevant benefit on hyperactivity was obtained over a 2-week period, as rated by teachers and parents on the hyperactivity subscale of the Aberrant Behavior Checklist (ABC) (14). Nonetheless, the effects of MPH do not appear to be age-dependent. Data on the effects of MPH on impulsivity and social or communication skills are currently insufficient (14).

### Safety and tolerability of methylphenidate

Among the twenty-eight studies reviewed, four clinical trials and four meta-analyses investigated the safety and tolerability of MPH (11, 13, 15, 18–22). To date, the available literature is unable to provide reliable data on the possible iatrogenic effects of MPH, both due to the lack of longitudinal studies in the sector and the immediate exclusion from the sample of children who showed early side effects after the test of the first dose.

Regarding the side effects produced by MPH, the meta-analysis conducted by Reichow et al. demonstrated that MPH is effective for the treatment of ADHD symptoms in children with Pervasive Developmental Disorders (PDD) (15). However, the review reports a significantly increased risk of side effects associated with MPH use in children with PDD compared to MPH use in children with ADHD alone (15). Notably, children with PDD treated with MPH, compared to the sample of children with ADHD alone, showed a greater increase (>15%) in the likelihood of experiencing both insomnia and decreased appetite with respect to abdominal pain, social withdrawal, emotional outbursts and irritability.

This analysis is supported by the prospective non-randomised observational cohort study conducted by Lilja and colleagues in 323 patients aged 6 to 17 years (22). The study evaluating the effect of different drugs (Methylphenidate, Dexamphetamine, Lisdexamphetamine, Atomoxetine and Guanfacine) in patients with ASD and ADHD, reported no significant differences in drug treatment effects between patients with ADHD and concurrently pronounced symptoms of ASD and ADHD patients without a high level of ASD symptoms. Moreover, no significant difference in the number of clinically significant adverse events reported between groups (22) has been reported.

For this reason, the study authors recommend a careful monitoring of MPH prescription in preschool children (15, 19). Furthermore, MPH appears to be better tolerated by children with higher cognitive functioning (IQ > 80) (15, 19).

A study by Pearson et al. on a sample of 24 children with a dual diagnosis of ASD-ADHD showed better sustained and selective attention after a treatment with a higher dose of MPH (18). These improvements were linear and dose-dependent. Furthermore, the results of a later study showed better inhibition ability and shorter reaction times in the study group, even at a lower dosage, compared to the control group of children not treated with MPH (18). This suggests that even a lower MPH dosage could have a positive effect on cognition.

This assertion is consistent with the findings of a study conducted by Kim et al., which underlines how the extended release of MPH might be more effective in reducing ADHD symptomatology (13).

Furthermore, the research group of Rodrigues et al. analysed all randomized controlled trials performed on participants under 25 years of age who were diagnosed with ASD (21). Such trials evaluated hyperactivity, impulsivity, and inattention following treatment with stimulants (MPH or amphetamines), atomoxetine, alpha-2 adrenergic receptor agonists, antipsychotics, tricyclic antidepressants, bupropion, modafinil, venlafaxine, or a combination of these (21). Specifically, the outcomes were compared with those of placebo and other behavioural therapies. Data were pooled using a random-effects model. The meta-analysis showed that MPH reduced hyperactivity, but that the drug was associated with a non-significant elevated risk of dropout due to adverse events (21). For the sake of completeness, it must be emphasized that the response rate to MPH intake varies from 50% to 60% in children with ASD-ADHD, while it appears higher (70%–80%) in children with the sole diagnosis of ADHD (11).

### Physiopathology and methylphenidate

Ten studies analysed the neurophysiopathology of the comorbidity of ASD and ADHD (3, 14, 17, 23–28). Both diseases share several common aspects, such as early onset, neurological and developmental delay, cognitive impairment, male predominance, and a strong genetic influence, and can therefore be envisioned on the same continuum (24, 25).

Family and twin studies suggest that both conditions might share a common genetic basis: between 50% and 72% of the genetic factors that lead to ASD and ADHD coincide (3, 17, 23). These genetic factors may alter the neurochemical balance of the brain and possibly impair the subject's executive functions, thus leading to difficulties in cognitive flexibility, planning, working memory, response inhibition, and selective, divided, alternating, and sustained attention (3, 23). Although these are considered some of the central aspects of ADHD aetiology, recent findings have also shown alterations in these domains in ASD patients, thus strengthening the hypothesis of a common basis of the two conditions (23). Furthermore, as argued by Willcutt et al., both diseases show similar impairments of the fronto-striatal and fronto-parietal neural circuits (29).

Different tools, such as quantitative EEG (qEEG), functional magnetic resonance (fMRI), and functional near-infrared spectroscopy (fNIRS), have been used to identify the physiopathological markers and cortical networks functionally involved in the symptomatology of the comorbidity between ASD and ADHD. For example, fNIRS tracks the cerebral haemodynamic response during the execution of specific tasks (30). Therefore, it is believed to be a practical tool that can provide significant data without requiring invasive procedures. A study carried out by Sutoko et al. highlighted a major difference in the cerebral activation of children with ADHD and ASD compared to neurotypical children (27). In this study, neurotypical subjects showed lower levels of oxygenated haemoglobin (Hb) in the right inferior and middle frontal gyri (IFG/MFG), areas responsible for inhibition control. In contrast, children with ASD and ADHD show an hypoactivation of the right prefrontal cortex (PFC) during the execution of go/no-go tasks, facial and gaze recognition, and tasks that require the identification of someone else's mental state (27). To support these findings, Anagnostou identified the PFC as the most affected network in children with ASD and ADHD (26). Usually involved in complex activities and social tasks, the PFC appears hypoactive in children with this condition. Hypoactivation of the prefrontal regions, but not the parietal regions, would explain the reason behind a reduction in attention-related deficits but not hyperkinesia after treatment with MPH. This would also explain why MPH does not seem to lead to significant improvements in ASD symptoms (26).

Overall, most clinical studies have considered MPH as a viable option for the treatment of symptoms of hyperactivity and inattentiveness in children with an ASD-ADHD dual diagnosis. In addition, MPH appears to have a significantly high affinity to dopamine receptor, the main neurotransmitter involved in prefrontal striatal functioning (27, 28).

Finally, from a neuropsychological point of view, there seems to be an association between Intellectual Quotient (IQ), level of autism and the pharmacological effect of psychostimulants. Subjects with better cognitive resources performance and with a higher level of autism (on the ADOS scale) seem to respond significantly better to drug therapy (14).

## Discussion

In this article, we presented a comprehensive review of the current literature on the efficacy of MPH for the treatment of children and adolescents with ADHD and ASD.

The main findings from this mini-review refer to subjects presenting ASD-ADHD not associated with other genetic or epileptic disorders, such as FXS, Tuberous Sclerosis, Kleefstra Syndrome or Angelman Syndrome. Specifically, 28 articles on PubMed and PsycArticle were used to examine the efficacy of MPH in children presenting with dual ASD-ADHD diagnoses (29).

From a clinical point of view, ASD appears as a neurodevelopmental disorder that requires multidisciplinary intervention and often shows symptoms comparable to those of ADHD.

There is evidence that ADHD is highly prevalent in ASD: 24%–84% of individuals diagnosed with ASD may also meet the criteria for ADHD (7, 30).

Both clinical trials and meta-analyses agree that to date there is no solid evidence to support recommendations on pharmacological treatments for complex diseases such as ASD-ADHD; however, this studies agree that non-pharmacological interventions focusing on environmental and behavioural factors could partially improve the symptomatology, although they do not appear to have long-term effects on the quality of life of the individuals (14, 15, 21, 31–33).

As reported in several studies, pharmacotherapy should therefore be included in a systemic intervention, which needs to consider multiple factors (14, 15, 34).

According to the current guidelines, such individuals need to follow the same treatment prescribed to patients with a sole ADHD diagnosis (35).

Currently, four classes of drugs have been identified for the treatment of a variety of symptoms in ASD: antidepressants, psychostimulants, antipsychotics, and melatonin (1). Since 2010, the prescription and intake of psychotropic drugs in children and adolescents with ASD have increased, especially with regard to psychostimulants and melatonin (Johansson et al., 2020). The use of prescribed drugs varies according to psychiatric comorbidity, symptomatology, age, and sex (Johansson et al., 2020; 13). However, interestingly, children diagnosed with ASD comorbid with ADHD appear to be less likely to initiate drug treatment than children diagnosed with ADHD alone. This event is thought to depend on the lack of clear guidelines or indications for the treatment of this comorbidity and this could also be related to the observed lower response rate (36).

Several studies suggest that the use of MPH for the treatment of ADHD symptoms in children with ASD-ADHD fosters cognitive and behavioural improvements (18). Such improvements relate to sustained attention, selective attention, and impulsivity/inhibition (37). All this, of course, has an impact on the social relationships and quality of life of these children.

However, a recent study by Lilja et al. highlights that the effect of stimulant drugs used for ADHD patients alone is lower in ADHD patients with concomitant ASD symptoms (22). Furthermore, the results support that ADHD patients with ASD symptoms experienced significantly more side effects than ADHD patients without ASD symptoms.

Unfortunately, the studies analysed do not provide indications on the long-term effects of therapy in social relationships, behavioural and cognitive response. For this reason, further follow-up studies are needed; nevertheless, it should be considered that the use of psychostimulant drugs in the diagnosis of ASD-ADHD aims at reducing specific parameters such as inattention or hyperkinesis that partly affect social relationships but which are not the determinants of relationship problems in ASD disorder.

As regards the use of rating scales (Table 1), most of the studies adopted the ADOS and ADI-R scales for the diagnosis of ASD and the CONNERS scale for ADHD (38–42). Furthermore, for the behavioural assessment after MPH administration in children or adolescents diagnosed with ASD-ADHD, the Autism Behavior Check List was used (43–52).

The main strength of this mini review has been a careful research methodology by adopting the *P*.I.C.O. model, but our conclusions are limited by the number and quality of the existing published studies.
